# Know thy cells: commonly used triple-negative human breast cancer cell lines carry mutations in RAS and effectors

**DOI:** 10.1186/s13058-022-01538-8

**Published:** 2022-06-27

**Authors:** Kay-Uwe Wagner

**Affiliations:** Department of Oncology, School of Medicine and Tumor Biology Program, Barbara Ann Karmanos Cancer Institute, Wayne State University, 4100 John R, Mail Code EL01TM, Detroit, MI 48201 USA

A new article by Tang and coworkers [1] reported that Neuropilin‑1 (NRP1) may play a role in tumor stem cell features and the development of claudin-low breast cancer, which, depending on the cell(s)-of-origin and developmental trajectories, could be defined as a molecular subtype or as a phenotype of other intrinsic subtypes [2]. The work further highlights the significance of oncogenic RAS/MAP kinase signaling that has been reported earlier to be a recurrent feature across claudin-low breast tumors [3]. Discussing the potential implications of their work, Tang and colleagues stated that mutations in RAS/MAPK are infrequent in human breast cancers and that NRP1 expression might be the crucial link for the activation of RAS through receptor tyrosine kinases (RTKs). Based on the experimental work presented, such a conclusion should be taken with caution due to the simple fact that three of the four claudin-low tumor cell lines used in this study carry mutations in KRAS (MDA-MB-231) and HRAS (SUM159PT, Hs578T) [4]. Moreover, all three cell lines have mutations in RAS effector pathways. Specifically, MDA-MB-231, which is the most commonly used triple-negative breast cancer cell line model (over 18,000 citations in PubMed), carries mutations in *BRAF* and *NF1* (COSMIC). Oncogenic RAS signaling is an important genetic element for the transformation of primary human cells [5], and it should be noted that many human cancer cell line models that are being employed in breast cancer research carry mutations in the RAS pathway, including MCF10AT (HRAS) and its derivative MCF10DCIS.com (Cellosaurus) as well as most de novo transformed HMECs with RAS^G12D^ from various laboratories such as those of Drs. Weinberg, Band, and Eaves, [6–8]. Beyond the events associated with neoplastic transformation, a high activation of RAS signaling specifically in claudin-low breast cancers seems to be a molecular determinant for the mesenchymal characteristics. Recent work from our team has demonstrated that oncogenic RAS signaling promotes cellular plasticity and the genesis of primary claudin-low mammary tumors in genetically engineered mice [9]. The persistent activation of this pathway seems to be critical for the preservation of the mesenchymal features that define this molecular tumor subtype.

Despite gene expression data from human tumors and experimental evidence using genetic models that support important roles of oncogenic RAS/MAPK signaling in mammary cancer initiation and progression, the importance of this pathway is often diminished by comments about the infrequent occurrence of RAS mutations in primary human breast cancers. It should be noted that breast cancer is not a single disease, and the rates of mutations vary among molecular subtypes and whether the underlying cause of a malignant tumor is hereditary or sporadic. Pathogenic mutations in BRCA1 or BRCA2 are not substantially more common than RAS mutations across all cases, and when it comes to hereditary cancers, patients with certain RASopathies are at greater risk of developing early-onset breast cancer [10]. Studies have shown that genetic alterations in RAS and MAP kinases, as well as loss of NF1, occur more often in TNBCs [11]. Additionally, RAS/MAPK pathway alterations were reported to be enriched in residual breast cancers following chemotherapy [12], and it may therefore not be surprising that many established TNBC cell lines that were derived from distant metastases, including those used in the study by Tang et al., have mutations in KRAS or HRAS and their downstream effectors. While these mutations greatly amplify the strength and duration of the signaling pathway, the functionality of mutant RAS may require upstream activators. Hence, the specific upregulation of NRP1 in claudin-low breast cancer and its potential role in RTK signaling in the new report by Tang et al. might be important, but the presence of multiple driver mutations within the pathway of the particular cellular models must be taken into consideration to accurately assess the contribution of NRP1 as a signaling modulator for RAS and MAP kinases and the cellular characteristics that they instigate.

## Data Availability

Not applicable.

## References

[1] Tang YH, Rockstroh A, Sokolowski KA, Lynam L-R, Lehman M, Thompson EW, Gregory PA, Nelson CC, Volpert M, Hollier BG (2022). Neuropilin-1 is over-expressed in claudin-low breast cancer and promotes tumor progression through acquisition of stem cell characteristics and RAS/MAPK pathway activation. Breast Cancer Res.

[2] Fougner C, Bergholtz H, Norum JH, Sorlie T (2020). Re-definition of claudin-low as a breast cancer phenotype. Nat Commun.

[3] Pommier RM, Sanlaville A, Tonon L, Kielbassa J, Thomas E, Ferrari A, Sertier AS, Hollande F, Martinez P, Tissier A (2020). Comprehensive characterization of claudin-low breast tumors reflects the impact of the cell-of-origin on cancer evolution. Nat Commun.

[4] Hollestelle A, Elstrodt F, Nagel JH, Kallemeijn WW, Schutte M (2007). Phosphatidylinositol-3-OH kinase or RAS pathway mutations in human breast cancer cell lines. Mol Cancer Res.

[5] Hahn WC, Counter CM, Lundberg AS, Beijersbergen RL, Brooks MW, Weinberg RA (1999). Creation of human tumour cells with defined genetic elements. Nature.

[6] Elenbaas B, Spirio L, Koerner F, Fleming MD, Zimonjic DB, Donaher JL, Popescu NC, Hahn WC, Weinberg RA (2001). Human breast cancer cells generated by oncogenic transformation of primary mammary epithelial cells. Genes Dev.

[7] Bhagirath D, Zhao X, West WW, Qiu F, Band H, Band V (2015). Cell type of origin as well as genetic alterations contribute to breast cancer phenotypes. Oncotarget.

[8] Nguyen LV, Pellacani D, Lefort S, Kannan N, Osako T, Makarem M, Cox CL, Kennedy W, Beer P, Carles A (2015). Barcoding reveals complex clonal dynamics of de novo transformed human mammary cells. Nature.

[9] Rädler PD, Wehde BL, Triplett AA, Shrestha H, Shepherd JH, Pfefferle AD, Rui H, Cardiff RD, Perou CM, Wagner KU (2021). Highly metastatic claudin-low mammary cancers can originate from luminal epithelial cells. Nat Commun.

[10] Sharif S, Moran A, Huson SM, Iddenden R, Shenton A, Howard E, Evans DG (2007). Women with neurofibromatosis 1 are at a moderately increased risk of developing breast cancer and should be considered for early screening. J Med Genet.

[11] Network TCGA (2012). Comprehensive molecular portraits of human breast tumours. Nature.

[12] Balko JM, Giltnane JM, Wang K, Schwarz LJ, Young CD, Cook RS, Owens P, Sanders ME, Kuba MG, Sánchez V (2014). Molecular profiling of the residual disease of triple-negative breast cancers after neoadjuvant chemotherapy identifies actionable therapeutic targets. Cancer Discov.

